# 27-Hydroxycholesterol, The Estrogen Receptor Modulator, Alters DNA Methylation in Breast Cancer

**DOI:** 10.3389/fendo.2022.783823

**Published:** 2022-03-10

**Authors:** Ravindran Vini, Arumugam Rajavelu, Sreeja Sreeharshan

**Affiliations:** ^1^ Cancer Research Program, Rajiv Gandhi Centre for Biotechnology (RGCB), Thiruvananthapuram, India; ^2^ Pathogen Biology, Rajiv Gandhi Centre for Biotechnology (RGCB), Thiruvananthapuram, India; ^3^ Department of Biotechnology, Bhupat & Jyoti Mehta School of Biosciences, Indian Institute of Technology, Chennai, India

**Keywords:** breast cancer, epigenetics, DNA methylation, 27-hydroxycholesterol, estrogen receptor alpha, liver X receptor (LXR)

## Abstract

27-hydroxycholesterol (27-HC) is the first known endogenous selective estrogen receptor modulator (SERM), and its elevation from normal levels is closely associated with breast cancer. A plethora of evidence suggests that aberrant epigenetic signatures in breast cancer cells can result in differential responses to various chemotherapeutics and often leads to the development of resistant cancer cells. Such aberrant epigenetic changes are mostly dictated by the microenvironment. The local concentration of oxygen and metabolites in the microenvironment of breast cancer are known to influence the development of breast cancer. Hence, we hypothesized that 27-HC, an oxysterol, which has been shown to induce breast cancer progression *via* estrogen receptor alpha (ERα) and liver X receptor (LXR) and by modulating immune cells, may also induce epigenetic changes. For deciphering the same, we treated the estrogen receptor-positive cells with 27-HC and identified DNA hypermethylation on a subset of genes by performing DNA bisulfite sequencing. The genes that showed significant DNA hypermethylation were *phosphatidylserine synthase 2 (PTDSS2), MIR613, indoleamine 2,3-dioxygenase 1 (IDO1), thyroid hormone receptor alpha (THRA), dystrotelin (DTYN),* and *mesoderm induction early response 1, family member 3 (MIER)*. Furthermore, we found that 27-HC weakens the DNMT3B association with the ERα in MCF-7 cells. This study reports that 27-HC induces aberrant DNA methylation changes on the promoters of a subset of genes through modulation of ERα and DNMT3B complexes to induce the local DNA methylation changes, which may dictate drug responses and breast cancer development.

## Introduction

The 27-hydroxycholesterol (27-HC) is an oxysterol and is the first known endogenous selective estrogen receptor modulator (SERM) (1) that has been proven to enhance estrogen receptor (ER)-positive breast cancer (BC) proliferation *via* estrogen receptor alpha (ERα) (2). The ER-mediated proliferative action of 27-HC was found to be predominant over the growth inhibitory actions of the liver X receptors (LXRs), which could be further modulated by cellular and microenvironment factors (2). The proliferative effects of 27-HC have been demonstrated in MCF-7 xenograft model (3, 4). At the clinical level, the elevated tumoral mRNA expression of CYP7B1, the key enzyme responsible for the catabolism of 27-HC, is associated with increased recurrence-free survival (5). Also, The Cancer Genome Atlas (TCGA) data analysis showed that CYP27A1 expression is almost the same in breast tumors compared to normal breast tissues, while CYP7B1 expression is significantly lower (6). Interestingly, CYP7B1 hypermethylation and the recruitment of monocytes to breast tissue enhances accumulation of 27-HC (7). It is also observed that serum 27-HC concentration does not correlate with its levels in breast tumor unlike in normal breast tissue, pointing to a likely association of dysregulation of 27-HC metabolism in breast cancer cells (2). Though a study demonstrates that the elevated levels of circulating 27-HC are associated with a lower risk of breast cancer in postmenopausal women (8), a recent study shows that intratumoral CYP27A1 expression is associated with unfavorable tumor characteristics (9). It is interesting to note that it is not the circulating 27-HC but rather intratumoral levels that give a better picture of its tumorigenicity.

Studies suggest that epigenetic changes, particularly DNA methylation, are highly deregulated in many cancer cells (10, 11). Neoplasms often exhibit global DNA hypomethylation that is accompanied by promoter hypermethylation that may include tumor suppressor genes (12). This variation along with changes in transcriptional factors involved in cell differentiation and stem cell maintenance can also initiate tumor development and aid in the growth and survival of cancer cells (12). Often, epigenetic changes such as DNA methylation and histone lysine acetylation and methylation in the cells are influenced by local environment stimuli (13).

Estrogen is known to drive ER-positive breast cancer and has been shown to alter the global DNA methylation pattern, histone modification, and microRNA expression during neoplastic transformation of mammary glands (14). The DNA methylation patterns and chromatin marks dictate the accessibility to chromatin remodelers and transcriptional factors to estrogen-responsive regions and thus define the endocrine response of estrogen (15–17) and SERMs (18) in each tissue. It has been recently shown that 3D epigenome remodeling is a key mechanism associated with endocrine resistance that consists of aberrant DNA methylation and differential ER-bound enhancer–promoter interactions (19–21).

The 27-HC, being an endogenous SERM, could probably alter endocrine responses and/or bring differential epigenetic signatures to deregulate gene expression. There is ample evidence showing aberrant DNA methylome signatures in breast cancer cells (21), but it is unknown whether 27-HC mediates epigenetic alteration in breast cancer cells. In the current study, we have identified DNA hypermethylation on subsets of genes in 27-HC-treated breast cancer cells. Furthermore, we studied the consequences of two genes and found that 27-HC-mediated hypermethylation at *PTDSS2* leads to transcriptional downregulation, whereas hypermethylation at the promoter of *MIR613* leads to biogenesis of miR-613. In addition, we have found that 27-HC reduces DNMT3B association with the ERα. The DNA hypermethylation on subsets of genes might contribute to cancer progression in addition to altering immune responses and increasing cell proliferation *via* ERα and metastasis *via* LXR. The global DNA methylome changes reported in breast cancer cells could be mediated by a multifactorial event, and 27-HC is one of the molecules that could contribute to the development of aberrant DNA methylomes and carcinogenesis.

## Experimental Procedures

### Cell Lines and Their Maintenance

MCF-7 and MDA-MB 231 cells were maintained in Dulbecco’s Modified Eagle Medium (DMEM) supplemented with 10% fetal bovine serum (FBS) and 1% penicillin and streptomycin (HiMedia, Cat # A002A) at 37°C with 5% CO_2_. The cells were always treated with phenol red less medium containing 5% charcoal-treated serum before proceeding with 27-HC and Dimethylsulfoxide (DMSO) treatment.

### Genomic DNA Isolation

The MCF-7 cells were seeded at a density of 10^6^ cells, followed by treatment with 5% charcoal-treated serum for 48 h, then cells were treated with 1 µM 27-HC or DMSO as vehicle control for 72 h. Furthermore, the cells were collected and washed with 1× PBS. The genomic DNA isolation was carried out using AllPrep DNA/RNA Mini kit (Qiagen Cat No. 80204) according to the manufacturer’s instructions. The quality and concentration of genomic DNA were measured using NanoDrop 2000.

### Genome-Wide Methylation Analysis

The genome-wide DNA methylation analysis was carried out using Infinium Methylation EPIC Bead Chip array using genomic DNA isolated from the MCF-7 cells treated with 27-HC (Test) and DMSO (Control). The DNA samples were prepared using Infinium HD Methylation assay kit. For bisulfite conversion of gDNA, the whole genome was enzymatically fragmented, and purified DNA was applied to the Bead Chips. During hybridization, the DNA molecules anneal to locus-specific DNA oligomers linked to individual bead types. The allele-specific primer annealing followed by single-base extension of the probes using dinitrophenyl- or biotin-labeled ddNTPs (A and T nucleotides are dinitrophenyl-labeled; C and G nucleotides are biotin-labeled). The unmethylated cytosines are chemically deaminated to uracil in the presence of sodium bisulfite, while methylated cytosines are refractory to the effects of bisulfite and remain cytosine. After extension, the array was fluorescently stained and scanned on Illumina iScan, and the intensities of the methylated and unmethylated bead types were measured.

### Data Analysis

The analysis of the Illumina array data was performed using Illumina Genome Studio software. The intensities of the methylated and unmethylated bead types were measured. The DNA methylation values were recorded as beta values. This value is a continuous variable between 0 and 1, representing the ratio of the intensity of the methylated bead type to the combined locus intensity, and was recorded for each locus in each sample *via* Genome Studio software. The proportion of CpG loci with significant differential methylation (beta value) was compared between control and test samples. The loci having significant differential DNA methylation were represented as a percentage of methylation in control vs. test samples. The genes nearest to differentially methylated loci were identified. Differentially methylated loci were classified according to their distance from the nearest gene as belonging to CpG islands, shelves, or shores.

### Promoter DNA Methylation Analysis by Sodium Bisulfite Conversion

The genomic DNA was isolated from MCF-7 cells, untreated or treated with 27-HC as described above. The bisulfite conversion of genomic DNA was carried out as described (22). Briefly, 187 µl of Solution I, sodium bisulfite solution (0.95 g NaHSO_3_ in 1.25 ml water and 325 µl 2 M NaOH), was added to 20 µl of genomic DNA and mixed well. To this, 73 µl of freshly prepared solution II [98.6 mg of 6-hydroxy-2,5,7,8-tetramethylchroman-2-carboxylic acid (Sigma, cat # 238813) is dissolved in 2 ml of dioxane] was added and mixed by pipetting. Following this, the mixture was incubated in a thermocycler in the following conditions: 15 min at 99°C, 30 min at 50°C, 5 min at 99°C, 1.5 h at 50°C, 5 min at 99°C, and 1.5 h at 50°C. This was followed by addition of 150 μl of sterile distilled water that was transferred to Ultracel YM-50 columns, placed on the collection tube, and then centrifuged at 14,000*g* for 15 min. The filtrate was discarded, and columns were washed with 1× TE buffer. Furthermore, 0.3 M NaOH (500 μl) was added to the column and was incubated at room temperature for 10 min to desulfonate the DNA. This was centrifuged and washed with 1× TE buffer. The column was placed upside down to which prewarmed 50 μl of 1× TE was added and incubated at room temperature for 1 min and then centrifuged at 1,000*g* for 10 min. The filtrate was collected, and the concentration of DNA was measured using NanoDrop 2000.

### PCR Amplification of Selected Genes From Bisulfite-Converted DNA

The bisulfite-converted DNA-specific primers were used to amplify the target promoter regions (**Table 1**). To amplify the selected target regions, 2 μl of the bisulfite-converted DNA was used as template for PCR in a 25-μl reaction mixture (1× PCR buffer, 1.5 mM MgCl_2_, 0.2 mM of each dNTP, 0.4 μM of each primer, and 2.5 U of HotStar Taq polymerase). The PCR was performed with the following program: 15 min at 95°C, 5 × (30 s at 94°C, 30 s at 60°C–50°C, 90 s at 72°C), 5 × (30 s at 94°C, 30 s at 55°C–45°C, 90 s at 72°C), 35 × (30 s at 94°C, 30 s at 50°C–40°C, 90 s at 72°C), 5 min at 72°C. After the PCR amplification, 5 μl of the PCR product is electrophoresed on an 8% Polyacrylamide gel electrophoresis (PAGE) gel and stained with gel red to visualize the PCR product under UV light. The PCR products were purified with illustra GFX™ PCR DNA clean-up kit (GE healthcare Cat No. 28-9034-70).

**Table 1:** List of primers.

List of primers used for bisulfite sequencing	
PTDSS2 S1	FP: TAGATGGTGGTTTGGGGTGA
	RP: CCCCAAAAAAAATAAAAATAAAA
PTDSS2 S2	FP: GGGTTTTGAAAGGTATTTTT
	RP: CTACTTCTAAACAAATACTACT
PTDSS2 S3	FP: GATTTTGTGTTTTTTTTTATTTT
	RP: ACCTCTAAATTTTAACTACC
PTDSS2 S4	FP: GGTTAGTGGATTTTAGATTTT
	RP: CTTTTTAATATCAACTCATCC
PTDSS2 S5	FP: TAGTTTAAGTATAGGGATTT
	RP: AAACACCCATTATAAAACAACC
MIR613 S1	FP: TGATTTTTGATTGGTTGAATTTATT
	RP: CAACCTCTACTTCTCAAACTCAAAC
MIR613 S3	FP: ATTTTGTTATTTAGGTTGGA
	RP: AATCCCAACACTTTAAAAAA
MIERS3	FP: TTTTATATTAGTTAGGAGGAGAAG
	RP: RACCCCTTATAAAACAAAAC
THRAS1	FP: GGGAGGATAGATGAGGTAATATAGG
	RP: CTCCTACCTTAACCTCCCAAAATAC
THRAS2	FP: TGTGAGTTTAGATTGAATATAGGTT
	RP: TCTTTCACAAAAAATACTAAAATTC
THRAS3	FP: TAGGGTTTTATTTTGAGAGT
	RP: AAAAATCTCCACCATACCAA
IDO1	FP: TGGTGTAATTTTGGTTTATTG
	RP: AACAAAATATCTCACTCCTAT
**Real-time PCR Primers**	
hsa-mir-613	FP: CGCAGAGGAATGTTCCTTCT
	RP: TCCAGTTTTTTTTTTTTTTTGGCA
PTDSS2	FP: CCTACAAGGGCAAGATGAAGAG
	RP: CAACAGGAACACCAGGATGAT
U6 snRNA	FP: CTCGCTTCGGCAGCACA
	RP: AACGCTTCACGAATTTGCGT
GAPDH	FP: TGCACCACCAACTGCTTAGC
	RP: GGCATGGACTGTGGTCATGAG

PTDSS2, Phosphatidylserine synthase 2 (S1-S5 represents different fragments of upstream of promoter sequence); MIR613 (S1,S2 represents different fragments of upstream of promoter sequence); MIER, mesoderm induction early response 1, family member 3; THRA, thyroid hormone receptor alpha (S1-S3 represents different fragments of upstream of promoter sequence); IDO1, indoleamine 2,3-dioxygenase 1.

### Sequencing of Bisulfite-Converted DNA

DNA sequencing reactions were carried out with the amplicons using the BigDye^®^ Terminator v3.1 cycle sequencing kit with specific primers (**Table 1**). The PCR conditions were as follows: one cycle at 95°C (10 s), followed by a series of 30 cycles that included treatment at 95°C for 30 s, at the annealing temperature (*Tm*) of the specific primer for 1 min, and at 65°C for 4 min. To the PCR reaction mix was added 2.5 µl of 125 mM EDTA and 10 µl of 3 M sodium acetate (pH: 4.8) and 100 µl of water and 260 µl of 100% ethanol and incubated for 20 min and centrifuged for 25 min at 11,000 rpm at room temperature. This was followed by 70% ethanol wash air-dried in the dark and sequenced (Applied Biosystems). The sequencing data were further analyzed for differential methylation.

### Analysis and Interpretation of Bisulfite Sequencing Data

The sequencing file was processed by using BioEdit Sequence Alignment Editor (Ibis Biosciences, Carlsbad, CA). The sequences were further analyzed and filtered for sequence identity, conversion rate, and clonal sequences with default parameters by using Bisulfite Sequencing DNA Methylation Analysis (BISMA) Software (23) and Bisulfite Sequencing Data Presentation and Compilation (BDPC) web interface (24). The BISMA program calculates the percentage of methylation with its default setting parameter.

### Co-Immunoprecipitation Assay

The MCF-7 cells were treated with either DMSO or 27-HC (1 µM) for 72 h, and the cells were lysed in Radioimmunoprecipitation assay (RIPA) buffer [50 mM Tris-HCl (pH 6.8), 0.1% sodium dodecyl sulfate (SDS), 0.5% NP40, 150 mM NaCl, 0.5% sodium deoxycholate]. The cell lysate was subjected to immunoprecipitation (IP) by incubating with either 1 µg of ChIP-grade ERα antibody (Diagenode C15100066-100) or 1 µg control mouse IgG (CST 5415s) antibody overnight at 4°C with gentle rotation. The protein A dynabeads (Invitrogen 10001D) were incubated with 3% bovine serum albumin (BSA) for 45 min in the rotator at 4°C. The IP samples were further incubated with the beads for 2 h and washed with the lysis buffer thrice. The beads were reconstituted in 1× PBS and denatured in 4× sample SDS-PAGE gel loading buffer heating at 95°C. The beads were carefully removed, and the supernatant samples were separated on 12% SDS-PAGE. The separated proteins were further transferred to polyvinylidene difluoride (PVDF) membrane and blocked with 5% BSA for an hour and washed with Tris Buffered Saline with Tween (TBST). The blot was incubated with primary antibody (anti-Dnmt3b #Ab2851, ERα antibody Diagenode C15100066) overnight at 4°C. The blots were further incubated with suitable secondary antibody for 90 min, and the membrane was developed using ECL reagent (Bio-Rad).

### RNA Isolation and Expression Analysis by qRT-PCR

Cells were seeded at a cell density of 1 × 10^5^ cells in 60-mm dishes. Total RNA was isolated by using TRIzol method, while the microRNA isolation was carried out using miRNeasy mini kit (Qiagen, cat # 217004). The RNA was quantified with NanoDrop. The miRNA gene expression was analyzed as described previously (25, 26). Briefly, 100 ng of microRNA was subjected to PolyA tailing and reverse transcription [10× Poly A Polymerase Reaction Buffer (0.5 M Tris-HCl, 2.5 M NaCl, 100 mM MgCl_2_, pH 8.1), 1 μl of 1 mM ATP, 1 µl 10 µM Real-time quantitative PCR (qRT-PCR) primer (**Table 1**), 1 µl dNTP mix, 0.5 µl (200 U/µl) M-MuLv Reverse transcriptase], 0.2 µl of *E. coli* Poly (A) polymerase 5,000 U/ml, 100 ng of microRNA, and DEPC water was mixed on ice. The mix was subjected to 42°C, followed by 95°C for 5 min. For mRNA, isolation was done by TRIzol method and the first strand cDNA synthesis with One Step RT-PCR Kit Ver2 (Cat. # RR055A, TAKARA). This was followed by qPCR using SYBR Premix Ex Taq II (TAKARA) and was performed according to manufacturers’ instructions in 7500 Real-Time PCR system (Applied Biosystems, Foster City, CA, USA). All the samples were assessed in technical triplicates, and the experiment was done in biological triplicates. The genes were normalized by using Glyceraldehyde 3-phosphate dehydrogenase (GAPDH) as endogenous control, and U6 SnRNA was used as microRNA endogenous control (**Table 1**).

### Analysis of Transcription Factor Binding Sites

Analysis of the putative transcription factor (TF) binding sites was carried out by using PROMO (27, 28) and TransMir (29, 30) along with a search to unveil the potential association between the differentially methylated CpG sites and TFs that may bind to the regulatory regions of *PTDSS2* and *MIR613*. The miRNA-centric network visual analytics platform that was used to analyze miRNA centric network and understand how the TFs and genes associated with the miRNA is connected using Backbone Algorithm. The Pathway Finder tool was used to study the connection between hsa-miR-613 and ESR1 and LXR, both of which are receptors of 27-HC.

## Results

### Treatment of MCF-7 Cells With 27-Hydroxycholesterol Induces Aberrant DNA Methylation Changes

**Figure 1:**
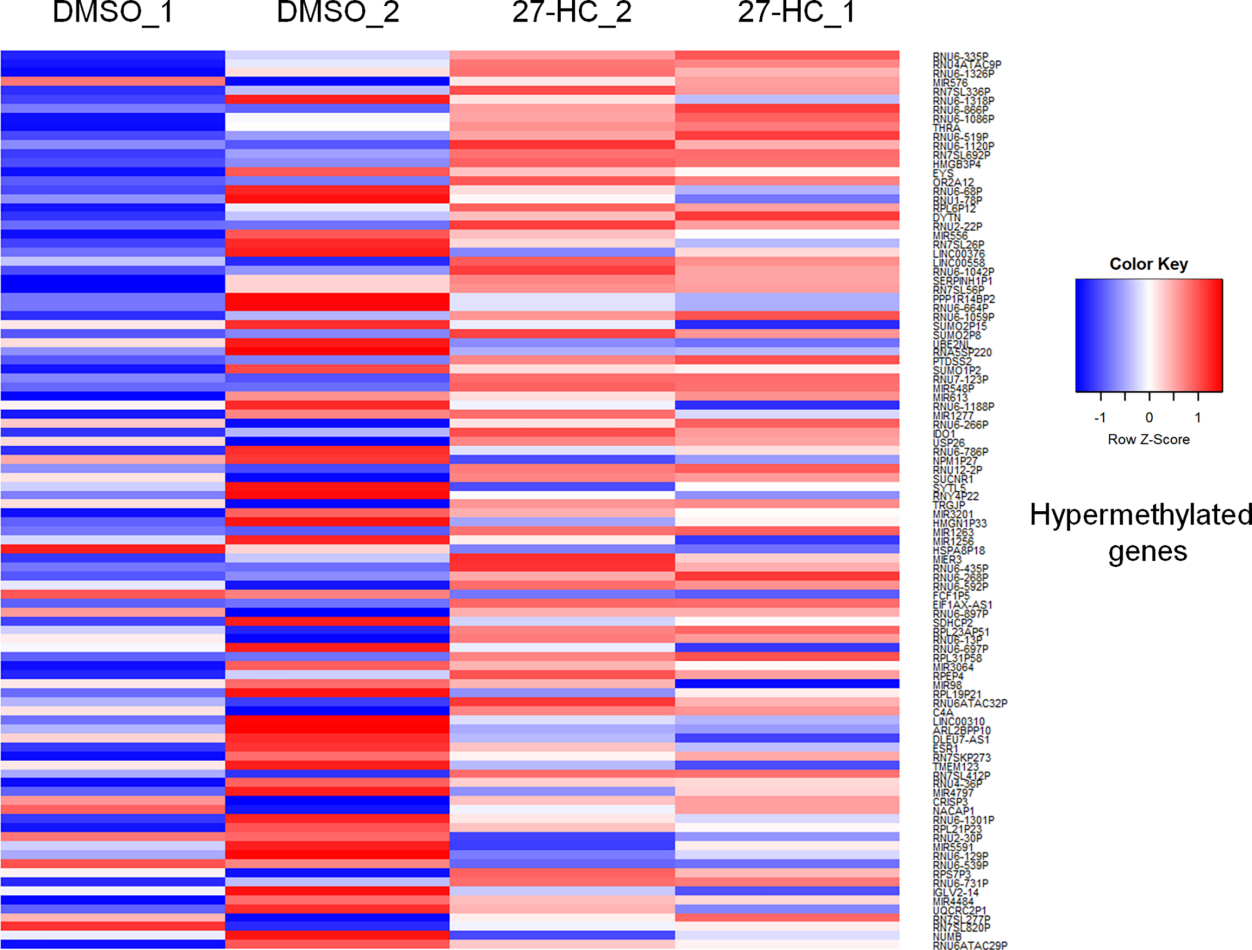
Identification of aberrant DNA methylation signatures: The heatmap represents the hypermethylation in 27-HC treated samples. The heatmaps were generated using beta values obtained from fluorescence intensity from the array scanning and the hypermethylated genes are with beta values greater that 0.5 fold change (treated > 0.5 compared to control) .The genes which showed significant DNA hypermethylation are phosphatidylserine synthase (*PTDSS2*), *mir613, indoleamine 2,3-Dioxygenase 1 (IDO1), thyroid hormone receptor alpha (THRA), Dystrotelin (DTYN), Mesoderm induction early response 1, family member 3 (MIER)*. The hypermethylated genes are shown in red color. The list of genes was taken for analysis from bisulfite sequencing (**Supplementary Data, S1**)

**Figure 2:**
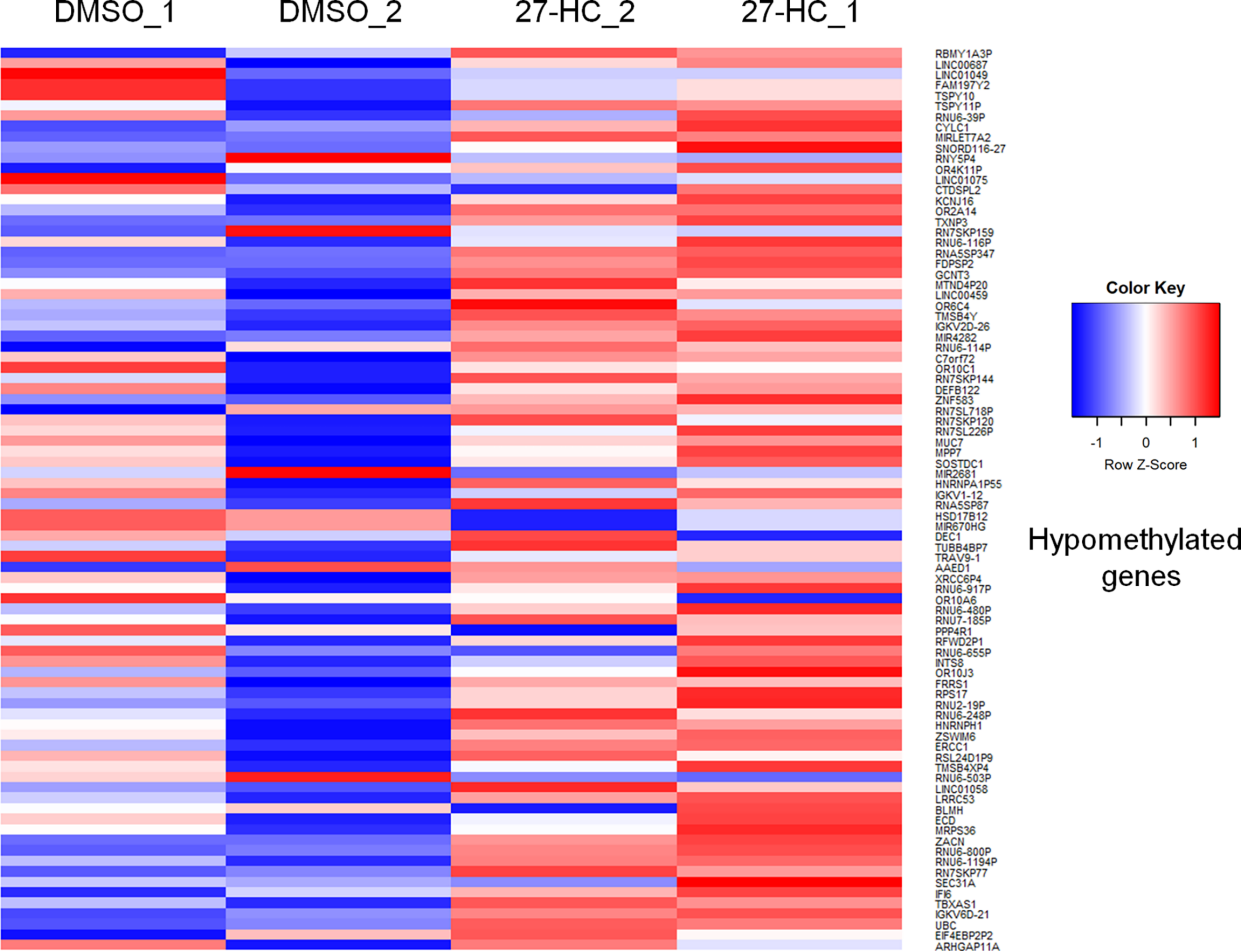
The heatmap represents the hypomethylation in 27-HC treated samples. The heatmaps were generated using beta values obtained from fluorescence intensity from the array scanning and the genes are considered hypomethylated when beta values are lesser than 0.5 (treated < 0.5 compared to control).

To evaluate whether 27-HC induces DNA methylation changes at the genome level, we performed methylation analysis with total DNA isolated from ER-positive breast cancer cells, MCF-7, after treatment with 1 μM of 27-HC for 72 h. This concentration was chosen as per our earlier study (31) and a report from another group (32), as 27-HC at this concentration, can significantly upregulate ERα-inducible targets and induces cell proliferation. The genes nearest the differentially methylated promoter were identified. The top 100 differentially methylated genes were selected (**Supplementary Figure S1**). Based on the beta values, hypermethylated and hypomethylated genes were selected and taken as input to generate the heatmaps. The genes were considered significantly hypermethylated when 27-HC-treated cells showed beta values greater than 0.5-fold change (treated >0.5 compared to control) and hypomethylated when less than 0.5 (treated <0.5 compared to control) (**Figures 1**, **2**). We found that there were a significant number of genes that showed hypermethylation upon 27-HC treatment when compared to DMSO control (**Figures 1**, **2**). However, among the genes that were hypomethylated, majority were found to be pseudogenes or non-coding genes (**Figures 1**, **2**). Hence, we focused on hypermethylated genes. Among those that showed significant DNA hypermethylation were *phosphatidylserine synthase 2 (PTDSS2), MIR613, indoleamine 2,3-dioxygenase 1 (IDO1), thyroid hormone receptor alpha (THRA), dystrotelin (DTYN),* and *mesoderm induction early response 1, family member 3 (MIER)* (**Figure 1**). The identified hypermethylated genes are directly and/or indirectly linked to cancer development and progression. From the results, it is evident that 27-HC induces aberrant DNA methylation changes with significant hypermethylation on a subset of genes in MCF-7 cells.

### Targeted DNA Methylation Analysis Confirms Promoter Hypermethylation

**Figure 3:**
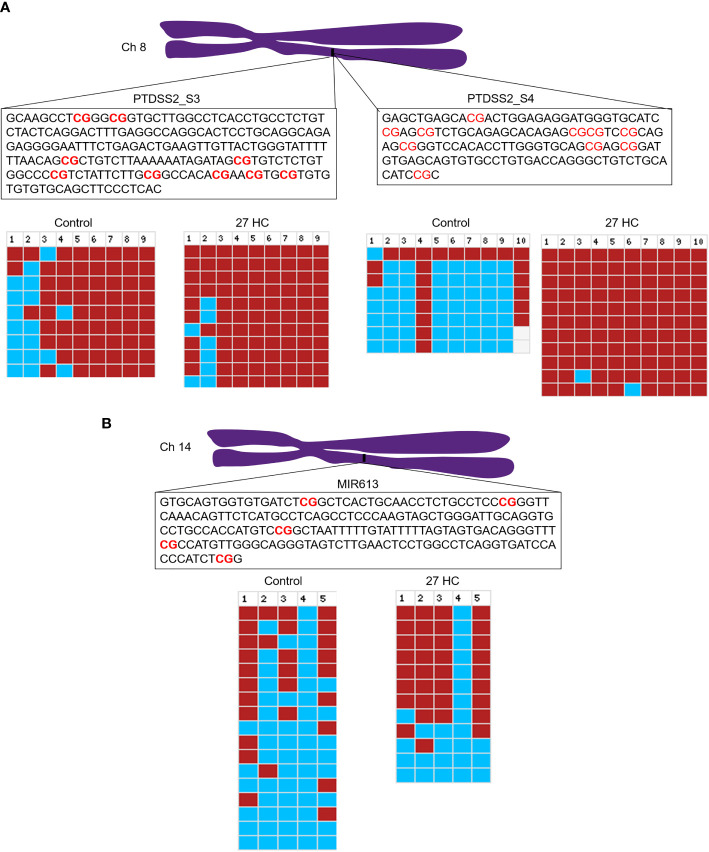
Validation of promoter DNA methylation by bisulfite sequencing: **(A)** Schematic representation of *PTDSS2* promoter region in the chromosome 8, and the boxes contain the sequences of two amplicons from *PTDSS2* gene promoter. The CpGs are highlighted in red. In the bisulfite sequencing analysis output from BISMA, the red box indicates methylated CpGs and the blue box indicates the unmethylated CpGs. Increased methylation is observed at the CpG 1, CpG 2, and CpG 3 in the *PTDSS2* amplicon 1 of 27-HC-treated cells than DMSO control. There is significantly increased methylation at the amplicon 2 in 27-HC-treated cells than DMSO control. Each row indicates individual clone, and the number at the top indicates the CpG sites of the corresponding amplicons. **(B)** The bisulfite sequencing analysis for *mir613* and location of its position on chromosome 14. The box contains the sequence of amplicon from the *mir613* promoter, and the CpG sites are highlighted in red. There is a significant level of hypermethylation at the CpG 2, 3, and 5 in 27-HC-treated cells than DMSO control. Each row indicates individual clone, and the number at the top indicates the CpG sites of the corresponding amplicon.

To validate the DNA hypermethylation observed in the Infinium array, we performed DNA bisulfite conversion and sequencing of the selected gene promoters. The promoter regions were identified based on the CpG island criteria, and the primers were designed covering CpG island which overlapped with TF binding sites (22). A list of DNA hypermethylated genes were selected for our analysis which were directly and/or indirectly associated with carcinogenesis (**Figure 1**). The bisulfite-converted DNA sequencing was performed using specific primers for *PTDSS2*, *mir613*, *IDO1, THRA, DTYN*, and *MIER*. Among these, the bisulfite sequencing analysis for *PTDSS2* and *mir613* confirmed the presence of DNA hypermethylation in 27-HC-treated cells compared with DMSO control (**Figures 3A, B**). We analyzed more than 10 clones for control and 27-HC-treated samples and observed that DNA hypermethylation is statistically significant (**Figures 3A, B**). We were not successful in obtaining good-quality sequencing reads for other selected genes.

### The 27-Hydroxycholesterol Regulates the Association of ERα With DNMT3B

**Figure 4:**
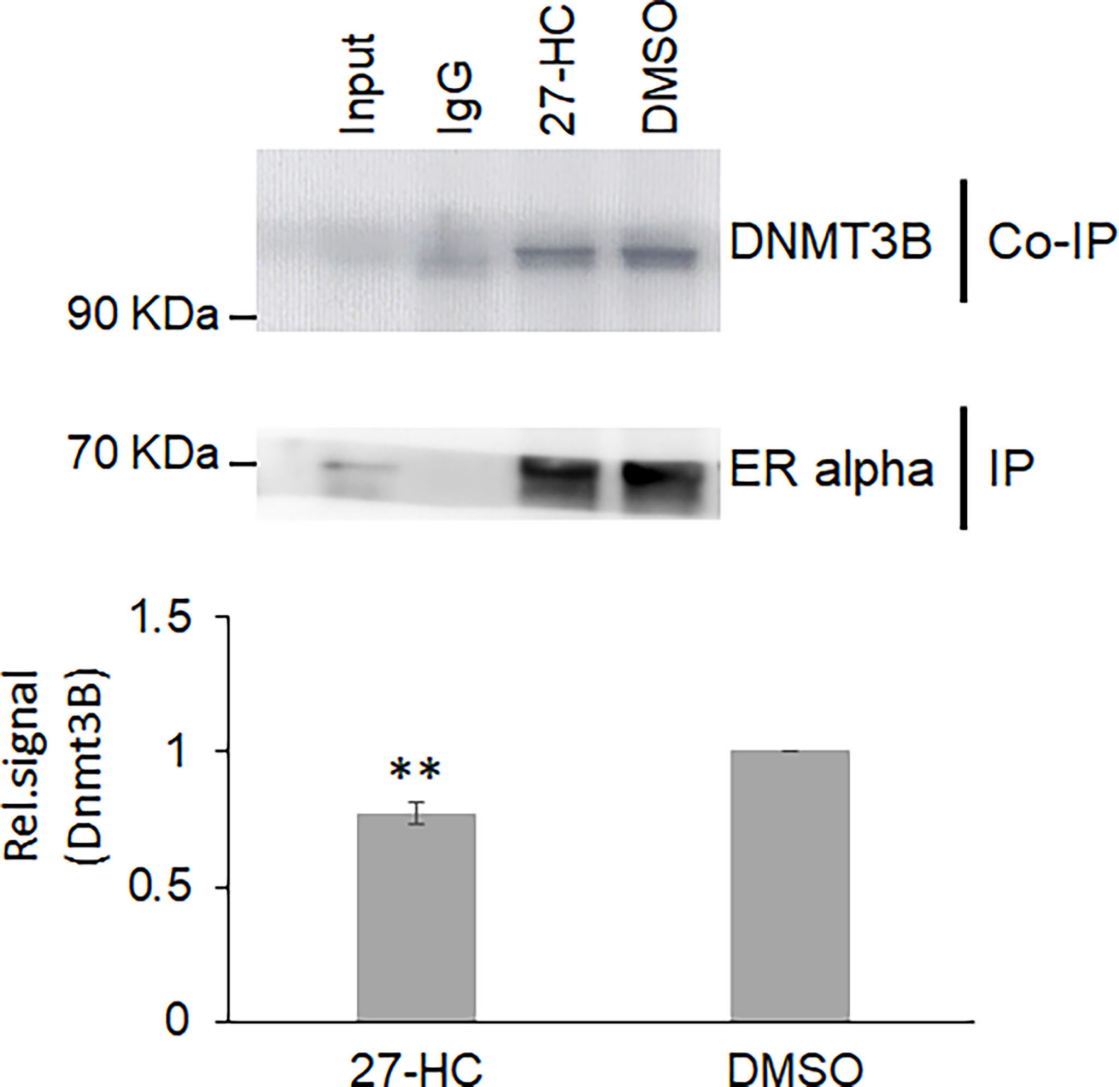
The 27-hydroxycholesterol (27-HC) weakens the DNMT3B association with estrogen receptor alpha (ERα): Co-immunoprecipitation (Co-IP) of ERα from DMSO- and 27-HC-treated cells has identified that the 27-HC causes a reduction in the association of DNMT3B with the ERα protein. The samples were separated on 12% gel and probed with the corresponding primary antibody. The ERα is used as a loading control. The bar graph represents the intensity of DNMT3B measured using ImageJ. The Co-IP assay was performed in triplicate, and the intensity of DNMT3B and ERα was measured using ImageJ. The normalized DNMT3B signal plotted and the error bar represent the standard error calculated from the biological triplicates. The p values were calculated using paired t-test, and the differences were considered significant at p values < 0.01 (**).

Next, we sought to understand the potential mechanisms of the 27-HC-mediated changes in DNA methylation in MCF-7 cells. The role of DNMT3B in breast cancer progression is well established, and the reports have shown elevated expression of DNMT3B protein when compared to other DNMTs such as DNMT3A and DNMT1 (33, 34). Moreover, it is known that DNMT3B interacts with the ERα and may contribute to breast cancer progression (35, 36). To understand the mechanism of action, we performed a co-immunoprecipitation assay of ERα protein to identify the status of DNMT3B association upon 27-HC treatment in MCF-7 cells. As reported earlier, we have found that ERα interacts with DNMT3B (34, 36) **(** **Figure 4**). Interestingly, we have found that 27-HC treatment of MCF-7 cells leads to reduced association of DNMT3B with the ERα (**Figure 4**). We speculate that the 27-HC-weakens the association of ERα with DNMTB which further may lead to free form of DNMT3B methyltransferases, causing DNA hypermethylation at the subsets of genes in MCF-7 cells. It is possible that ERα may contribute to preventing the aberrant activity of methyltransferase activity of DNMT3B.

### The 27-Hydroxycholesterol-Induced DNA Hypermethylation Transcriptionally Downregulates the *PTDSS2* Expression and Promotes the Biogenesis of *miR-613*

**Figure 5:**
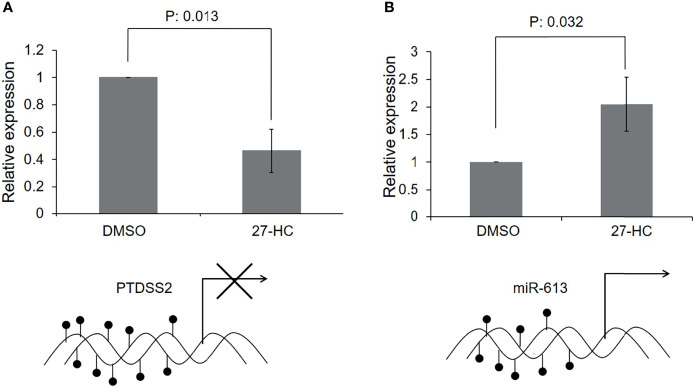
Transcriptional regulation of 27-hydroxycholesterol (27-HC)-mediated DNA hypermethylation: **(A)** The bar graph represents the qRT-PCR analysis for *PTDSS2* and has revealed the downregulation of its expression in 27-HC-treated cells. The GAPDH was used as normalization control, and the error bar represents the standard deviation of three independent replicates. The bottom scheme represents the hypermethylation of *PTDSS2* gene promoter leading to transcriptional repression. **(B)** The qRT-PCR analysis has revealed the biogenesis of miR-613 microRNA in 27-HC-treated cells. The *U6 miRNA* was used as internal normalization control to measure the relative expression of the *miR-613*, and the error bar represents the standard deviation of three independent replicates. The scheme at the bottom of the bar graph represents the hypermethylation of *mir-613* gene promoters, leading to transcriptional activation and biogenesis of miR-613. The p values were calculated using paired t-test, and the p values < 0.05 were considered significant.

Since there was hypermethylation at the promoters of the *PTDSS2* and *mir613* genes, we have analyzed the expression of these transcripts by qRT-PCR. We found that the *PTDSS2* gene expression was significantly downregulated upon 27-HC treatment, which suggests that the DNA hypermethylation at the promoters of *PTDSS2* transcriptionally downregulates the expression the genes (**Figure 5A**). Surprisingly, we observed significant upregulation of miR-613 microRNA in the 27-HC-treated cells when compared with that in DMSO control (**Figure 5B**). Although the DNA methylation mark at the gene promoter is known to be a strong gene repressor epigenetic mark, emerging reports show that the DNA methylation directs the miRNA biogenesis (37). Therefore, it is possible here that the promoter DNA hypermethylation is involved in transcriptional activation of *miR613* biogenesis in 27-HC-treated MCF-7 cells. The gain of DNA methylation events on these genes and its subsequent consequences are independent of each other and appear to be a general response to 27-HC treatment. Next, to confirm the role of ERα in 27-HC-mediated changes, we analyzed the *PTDSS2* expression upon 27-HC treatment in ERa receptor-negative MDA-MB 231 cells (**Supplementary Figure S1**). There was no statistically significant change (at p < 0.05) in the expression of *PTDSS2* upon 27-HC treatment in MDA-MB 231 cells, while we observed significant downregulation of *PTDSS2* in MCF-7 (**Figure 5** and **Supplementary Figure S1**). The results have clearly indicated that 27-HC regulated the expression of PTDSS2 is most likely *via* ERα in MCF-7 cells.

### Possible Mechanisms of DNA Hypermethylation-Mediated Regulation of PTDSS2 and miR-613 Genes

**Figure 6:**
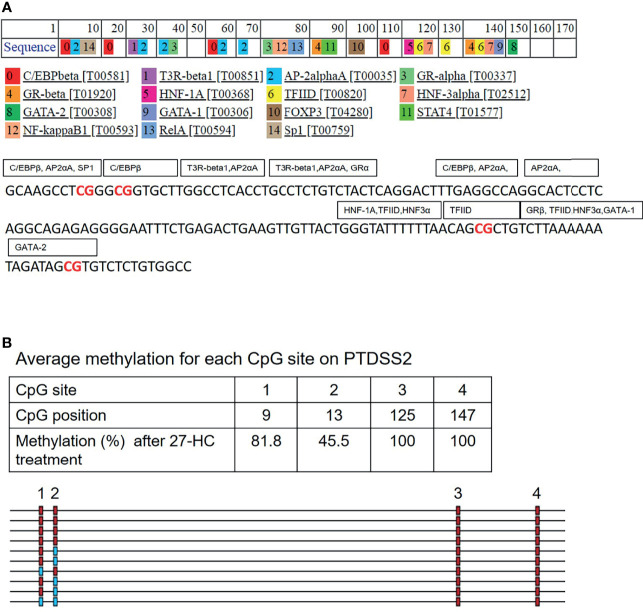
*In silico* analysis for transcription factor (TF) binding sites on *PTDSS2* promoter: **(A)** The list of predicted TFs for *PTDSS2* promoter sequence. The TFs are labeled in the box covering its putative binding sites on the promoter DNA sequence. The hypermethylated CpG sites are highlighted in red. **(B)** The output file from bisulfite sequencing analysis for *PTDSS2* gene promoter using BIMSA software and indicates the percentage of methylation at the top 4 CpG sites in the 27-hydroxycholesterol (27-HC)-treated samples.

The promoter DNA methylation mediates the gene suppression through either prevention of TF binding or recruitment of co-repressor molecules to the promoters. To get the mechanistic insights on the hypermethylation-mediated gene regulation, we performed *in silico* analysis to identify the potential TF that binds to the hypermethylated regions of *PTDSS2* gene promoter using PROMO (27, 28). It predicts the TF binding region on the promoter sequence from known binding sites (27). We analyzed *PTDSS2* promoter sequence with maximum matrix dissimilarity rate 5 and using other default parameters (27, 28). The identified putative TFs include ER-α, GATA-1, GATA-2 P53, GR-alpha, Sp1, FOXA1, NFκB FOXP3, and AP-2 (**Figure 6A**). The identified TFs are reported to be regulated by estrogen or ERs (38–47). Moreover, the predicted binding sites for TFs overlapped with the hypermethylated CpGs (**Figures 6A, B**). Also, it is known that methylation can affect the binding affinities of TF depending on the position of methylated CpGs within their binding motifs (48).

**Figure 7:**
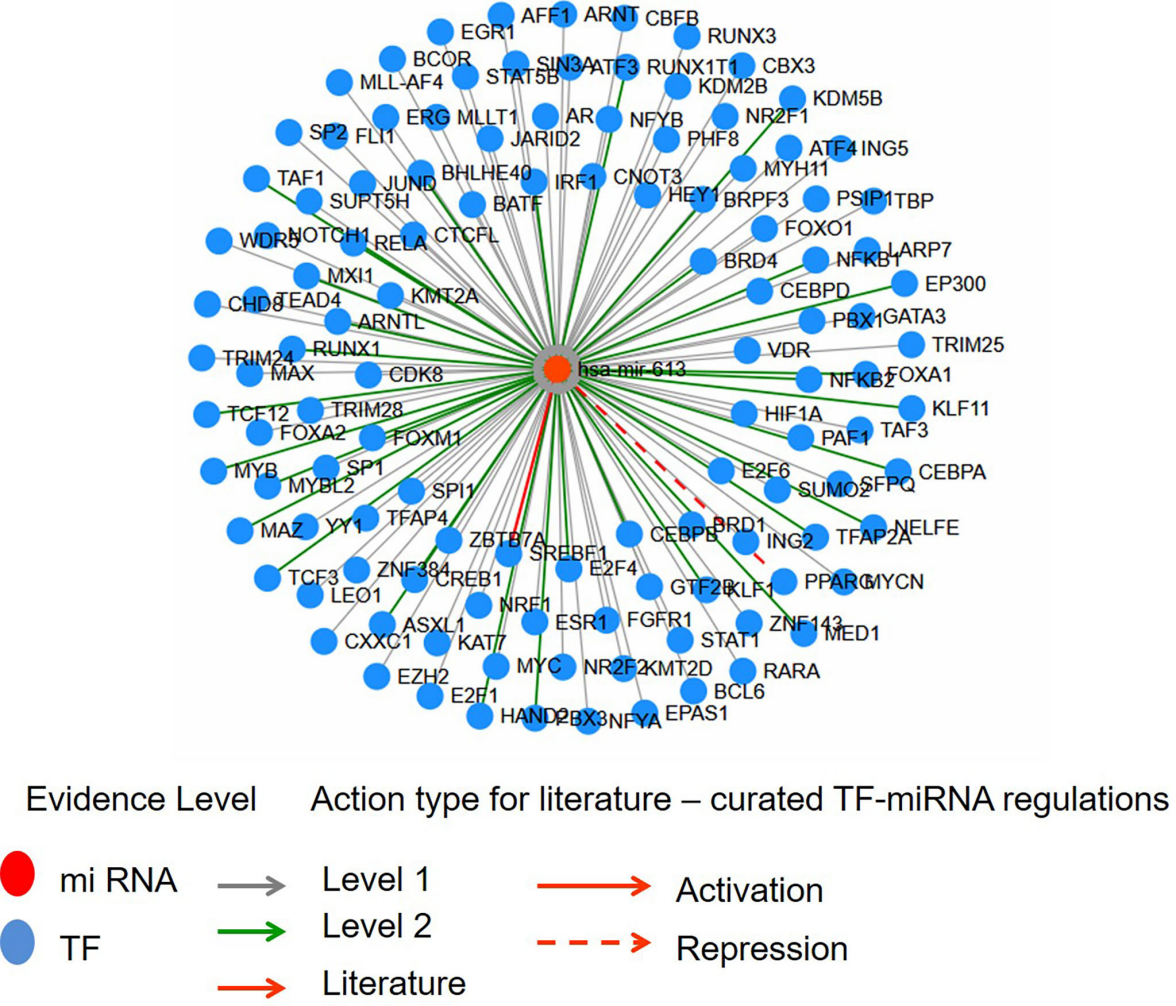
The interaction network of hsa-miR-613 with transcription factors (TFs) using Network module of TransMir. Level 1 is predicted, and level 2 is supported by high-throughput experimental data. TFs like ESR1, Sp1, FOXA1, GATA3, FOXM1, and AP1 are reported to be regulated by estrogen or 27-hydroxycholesterol (27-HC).

**Figure 8:**
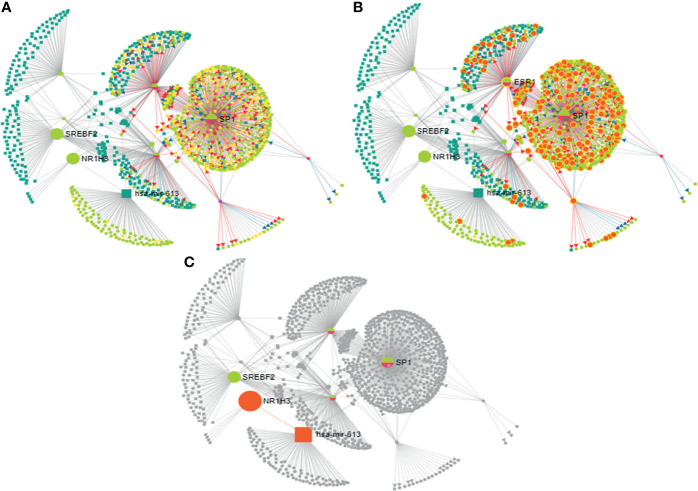
Analysis of mir-613 network using miRNA-centric network visual analytics platform: **(A)** Represents the network connecting hsa-mir-613 with the important transcription factors, miRNAs with the main node being NR1H3 (LXRα), SREBPF2, SP1, and ESR1 (ERα). **(B)** Enriched with function biological process: cell proliferation. **(C)** Represents the pathway connecting liver X receptor (LXR) and ESR1 (ERα) to hsa-mir-613.

The 27-HC treatment of MCF-7 cells leads to the biogenesis of miR-613, and hence, we studied the putative targets for miR-613 using TransMir network analysis. We selected a list of targets including Estrogen Receptor 1 (ESR1), specificity protein 1 (SP1), Forkhead Box A1 (FOXA1), GATA Binding Protein 3 (GATA3), forkhead box protein M1 (FOXM1) and Activator protein 1 (AP-1) that are regulated by either 27-HC or estrogen (42, 49) (**Figure 7**). Using a miRNA-centric network visual analytics platform, we analyzed the network and pathway link of miR613 using “backbone” algorithm with relevant genes ESR1 and LXR and the relevant TFs obtained using TransMir TFs (**Figures 8A–C**). The enrichment of the biological process “Cell proliferation” is represented in yellow and shows the pathways directly or indirectly involved in cell proliferation. We also analyzed the pathways connecting miR-613 to ERα and LXRα, both of which are receptors of 27-HC. These analyses have clearly indicated that the 27-HC-mediated aberrant promoter DNA methylation at these loci may alter the gene transcription probably inducing proliferative pathways. Taken together, the 27-HC molecule contributes to aberrant epigenetic changes on the subsets of genes in MCF-7 cells and further may aid in the proliferation or attaining invasive phenotypes of the cancer cells.

## Discussion

Aberrant DNA methylation is among the most common epigenetic alterations in neoplasia. It is known that breast cancer progression, development, and endocrine resistance arise as a result of accumulation of genetic and epigenetic changes in the genome (50). In mammary cells, estrogen deprivation (51) long-term exposure to estrogen (14) or SERMs (18, 52) leads to aberrant changes in DNA methylation. These epigenetic modifications can also result in endocrine resistance in ER-positive breast cancer. It is reported that aromatic inhibitor-resistant ER-positive breast cancer cells acquire the most aggressive phenotype, leading to stable upregulation of the entire cholesterol biosynthesis pathways that include genes involved in 27-HC biosynthesis (53). The 27-HC has been proven to induce breast cancer proliferation *via* ER and LXRs (3) and by modulating immune cells (54). Studies have shown that oxysterol and oxysterol sulfates like 25-hydroxycholesterol 3-sulfate are paired epigenetic regulators, agonists, and antagonists of DNA methyltransferases, indicating that their function of global regulation is through epigenetic modification (55). A recent report has shown that 27-HC stimulates the expression of maintenance DNA methyltransferase 1 (DNMT1) *via* Reactive oxygen species (ROS) pathway in MCF-7 cells (56). However, it is unknown whether 27-HC induces aberrant DNA methylation signatures in breast cancer cells. In the current study, we have employed Infinium methylome array, which covers maximum CpGs in the human genome to screen the 27-HC-induced aberrant DNA methylation changes in MCF-7 cells, and such changes may contribute to breast cancer cell proliferation. The DNA hypermethylation was observed on the subsets of genes in 27-HC-treated cells that include *PTDSS2*, *mir613*, *IDO1, THRA, DTYN*, and *MIER*. Among these, bisulfite sequencing has confirmed significant hypermethylation in *mir613* and *PTDSS2* (**Figure 1**). Hence, the expression of these two genes were analyzed upon 27-HC treatment.

The earlier reports have shown that the aberrant DNA methylation activity of DNMT3B is linked to the development and progression of breast cancer (35, 57). Nonetheless, the mechanisms of DNMT3B-mediated DNA hypermethylation remain obscure. The present study identifies that the DNMT3B interacts with ERα, and its association is modulated in the presence of 27-HC, a well-known agonist of ERα. The binding of 27-HC to ERα changes the conformation of the ERα protein (32), which probably leads to weakening ERα interaction with DNMT3B. We speculate that binding of ERα to DNMT3B is essential to regulate its activity in the cells, and reducing its association with partner proteins might lead to aberrant DNA methylation on a subset of genes.

The microRNA hsa-miR-613 is generally identified as a tumor suppressor in multiple cancers like bladder cancer (58), laryngeal squamous cell carcinoma, glioma cells, hepatocellular carcinoma, and osteosarcoma growth by downregulating oncogene expression and inhibiting the malignant potential of tumors (58). Here, we found hypermethylation at the promoter of miR-613 upon 27-HC treatment leads to upregulation of its expression. The miR-613 is known to directly target LXRα, the 27-HC receptor (59), through its specific miRNA response element (613MRE) within the LXRα 3′-untranslated region though the miRNA itself is induced upon LXR activation *via* SREBP-1c, an LXR target gene, thus mediating a feedback loop in its autoregulation (59), both of which are involved in cholesterol metabolism regulation. It has been reported that increased methylation in the flanking region of loci coding for miRNA results in increased miRNA biogenesis and is more probable to drive cancerous phenotypes (37). So, it is possible that 27-HC-mediated hypermethylation at the promoter of miR-613 and its biogenesis could aid in breast cancer progression, but detailed investigation is essential to understand the miRNA biogenesis in MCF-7 cells. TransMir network showed putative targets of miR-613 that are regulated by estrogen or ERs (42, 49), including ESR1, Sp1, FOXA1, GATA3, FOXM1, and AP1. Furthermore, miRNA-centric network visual analytics platform using the input given from data mining and our wet lab data shows an enrichment of genes involved in cell proliferation network, implying its importance in 27-HC-mediated cellular changes in breast cancer cell.

The PTDSS2 is involved in the synthesis of phosphatidylserine from phosphatidylethanolamine (PE) *via* a base-exchange reaction with serine in the mitochondria-associated membrane (MAM), a specific domain of endoplasmic reticulum (60). The PE acts as a feedback regulator of SREBP-mediated lipogenesis that leads to regulation of cholesterol homeostasis (61) that connects again to cholesterol regulation. Also, the phosphotidylserine externalization is a well-known signal for phagocytosis, and its aberrant expression is common in many cancers and considered as a potential therapeutic target (62, 63). But interestingly, it has been found that the *PTDSS2* gene has been deleted in few cancers (64). It has also been reported that this tumor suppressor gene is found to be deleted and is associated with other genomically colocalized HRAS (Harvey rat sarcoma viral oncogene homolog) pan cancer signature (65). The 27-HC-mediated transcriptional downregulation of *PTDSS2* is well correlated with the *PTDSS2* deletion phenotype observed in many cancers. *In silico* analysis of putative TFs that binds to the hypermethylated regions of *PTDSS2* gene promoter showed ER-α, GATA-1, GATA-2 P53, GR-alpha, Sp1, FOXA1, NFκB, FOXP3, and AP-2 that are regulated by estrogen or ERs (38–47). It is understood that methylation can affect binding affinities of TF depending on the position of methylated CpGs within their binding motifs (48). TFs like NFκB and AP-2 are known to repress from binding when there is methylation at binding sites (47, 48). Thus, this connotes that 27-HC-mediated promoter hypermethylation and downregulation of *PTDSS2* gene are most likely by affecting the local dynamics of TFs. Also, since we did not find any significant change in the expression of *PTDSS2* upon 27-HC treatment in MDA-MB 231 cells, we presume that the *PTDSS2* downregulation in MCF-7 is most likely *via* ERα. We also plan to investigate ER-α-specific effect in the upcoming work.

It is reported that epigenetic changes are linked to the development of breast cancer and can drive resistance acquisition and endocrine resistance. At this point, how *PTDSS2* and *miR-613* methylation aids in 27-HC-mediated breast cancer progression remains inexplicable. Observed DNA methylation changes in the presence of 27-HC might be due to multifactorial events; therefore, we aim to dissect the signaling cascade in future studies. Interestingly, the TF binding sites on *PTDSS2* hypermethylated region and TFs associated with miR-613 showed that many of these are closely associated with ERα and cancerous phenotypes. The methylations brought about by 27-HC exposure in these regions could possibly alter the gene transcriptions and their regulation. The 27-HC might also contribute to this cumulative effect in altering and bringing the tumorigenic changes *via* receptors and by epigenetic modifications. Further validation at tissue level and *in vivo* studies will help to ascertain its importance at the clinical level.

## Data Availability Statement

The original contributions presented in the study are included in the article/**Supplementary Material**. Sequence files have been deposited in Figshare via the DOI 10.6084/m9.figshare.18316553. Further inquiries can be directed to the corresponding authors.

## Author Contributions

RV, AR, and SS conceived the study and designed the experiments. AR and SS supervised the project. RV performed the research. RV, AR, and SS analyzed the data. RV, AR, and SS prepared the article draft. All the authors read and approved the final article.

## Funding

This work was supported by RGCB intramural support and ICMR extramural funding (RFC No. NCD/*Ad-hoc*/51/2021-22). RV is supported by ICMR Senior Research Fellowship, Government of India, and has registered her PhD under Kerala University.

## Conflict of Interest

The authors declare that the research was conducted in the absence of any commercial or financial relationships that could be construed as a potential conflict of interest.

## Publisher’s Note

All claims expressed in this article are solely those of the authors and do not necessarily represent those of their affiliated organizations, or those of the publisher, the editors and the reviewers. Any product that may be evaluated in this article, or claim that may be made by its manufacturer, is not guaranteed or endorsed by the publisher.

## Supplementary Material

Supplementary Figure S1 The ERα receptor negative breast cancer cells MDAMB was treated with 27-HC. The transcript expression analysis of PTDSS2 have confirmed that there are no changes (not statistically significant at p < 0.05) in the presence of 27-HC in MDA-MB 231 cells. The 27-HC induces DNA methylation changes and transcriptional downregulation of PTDSS2 through ERα receptor. The GAPDH expression was used to normalize the relative expression and the error bar represents the standard deviation of three independent replicates.

Supplementary Table 1 List of differentially methylated genes upon 27 HC treatment for 72 h in MCF-7 cells.
