# Supplementary figures and images for: 27-Hydroxycholesterol, The Estrogen Receptor Modulator, Alters DNA Methylation in Breast Cancer
Source: Front Endocrinol (Lausanne). 2022 Mar 10;13:783823. doi: 10.3389/fendo.2022.783823 (PMC8961300; doi:10.3389/fendo.2022.783823)

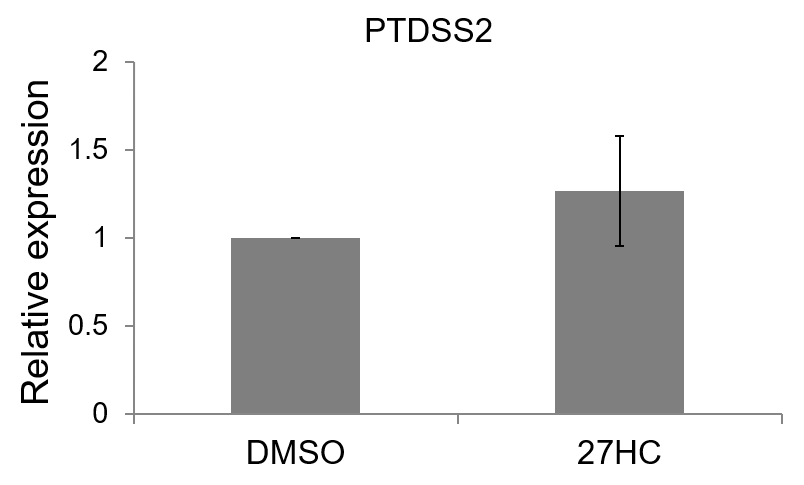

Supplement: Supplementary Figure S1 — The ERα receptor negative breast cancer cells MDAMB was treated with 27-HC. The transcript expression analysis of PTDSS2 have confirmed that there are no changes (not statistically significant at p < 0.05) in the presence of 27-HC in MDA-MB 231 cells. The 27-HC induces DNA methylation changes and transcriptional downregulation of PTDSS2 through ERα receptor. The GAPDH expression was used to normalize the relative expression and the error bar represents the standard deviation of three independent replicates. [file Image_1.tif]
